# Messing Up Texas?: A Re-Analysis of the Effects of Executions on Homicides

**DOI:** 10.1371/journal.pone.0138143

**Published:** 2015-09-23

**Authors:** Patrick T. Brandt, Tomislav V. Kovandzic

**Affiliations:** 1 Political Science, Economic, Political and Policy Sciences, University of Texas, Dallas, Richardson, Texas, United States of America; 2 Criminology, Economic, Political and Policy Sciences, University of Texas, Dallas, Richardson, Texas, United States of America; Mälardalen University, SWEDEN

## Abstract

Executions in Texas from 1994–2005 do not deter homicides, contrary to the results of Land et al. (2009). We find that using different models—based on pre-tests for unit roots that correct for earlier model misspecifications—one cannot reject the null hypothesis that executions do not lead to a change in homicides in Texas over this period. Using additional control variables, we show that variables such as the number of prisoners in Texas may drive the main drop in homicides over this period. Such conclusions however are highly sensitive to model specification decisions, calling into question the assumptions about fixed parameters and constant structural relationships. This means that using dynamic regressions to account for policy changes that may affect homicides need to be done with significant care and attention.

## Introduction

In the last decade there is a resurgence of academic studies estimating the possible deterrent effect of capital punishment on homicide rates [2–6] With few exceptions [7, 8] these recent deterrence studies employ non-experimental fixed-effects panel designs that span the period since the reinstatement of the death penalty in the U.S. after the 1976 Supreme Court decision in *Gregg v. Georgia*, and use ordinary least squares (OLS) or instrumental variables (IV) estimators. Joana Shepherd, author of several of these studies, summarizes the latest econometric findings in her congressional testimony as follows: “The modern studies have consistently shown that capital punishment has a strong deterrent effect, with each execution deterring between 3 and 18 murders” [9]. Another leading researcher in this area, Nai Mocan, the co-author of two of the recent panel studies is quoted in an Associated Press report about the robustness of the deterrent effects of executions, saying “Science does really draw a conclusion. It did. There is no question about it. The conclusion is there is a deterrent effect” [10].

Like their national, aggregate time-series predecessors [11], these pro-deterrence death penalty papers have been subjected to considerable academic scrutiny, with critics highlighting numerous conceptual and methodological problems. These include the failure of the research properly mitigating omitted variable bias (e.g., prison population growth), using possibly dubious deterrence ratio variables as proxies for potential homicide perpetrators’ perceptions of the expected costs of committing capital murder, assuming that executions are simultaneously determined (i.e., executions are endogenous events) through the application of the instrumental variables (IV) estimator (and even if that were the case using invalid and unreliable instrumental variables to instrument for execution risk), failing to correct standard errors for the presence of serial correlation, and sampling fragility of the results to alternative periods and outliers [12–16]. Despite several studies addressing these methodological shortcomings, the literature confirms critics’ suspicions that the recent findings were largely a byproduct of the aforementioned criticisms. When these issues are addressed, there is little or no robust empirical evidence of a significant relationship between the presence of the death penalty or increases in execution risk and the homicides rate. The pro-deterrence authors counter that the problems highlighted were either incorrect or inconsequential to their original conclusions [2, 17, 18].

Given this conflicting body of evidence, and the fact that many of the pro-deterrence death penalty research findings have found their way into the policy-making arena, it is not surprising then that the National Research Council (NRC) convened a panel of scholars to reconcile the latest scientific evidence on the deterrent effects of the death penalty. The NRC committee concluded that “research to date on the effect of capital punishment is not informative about whether capital punishment decreases, increases, or has no effect on homicide rates. Therefore, the committee recommends that these studies not be used to inform deliberations requiring judgments about the effect of the death penalty on homicide” [19].

**Fig 1:**
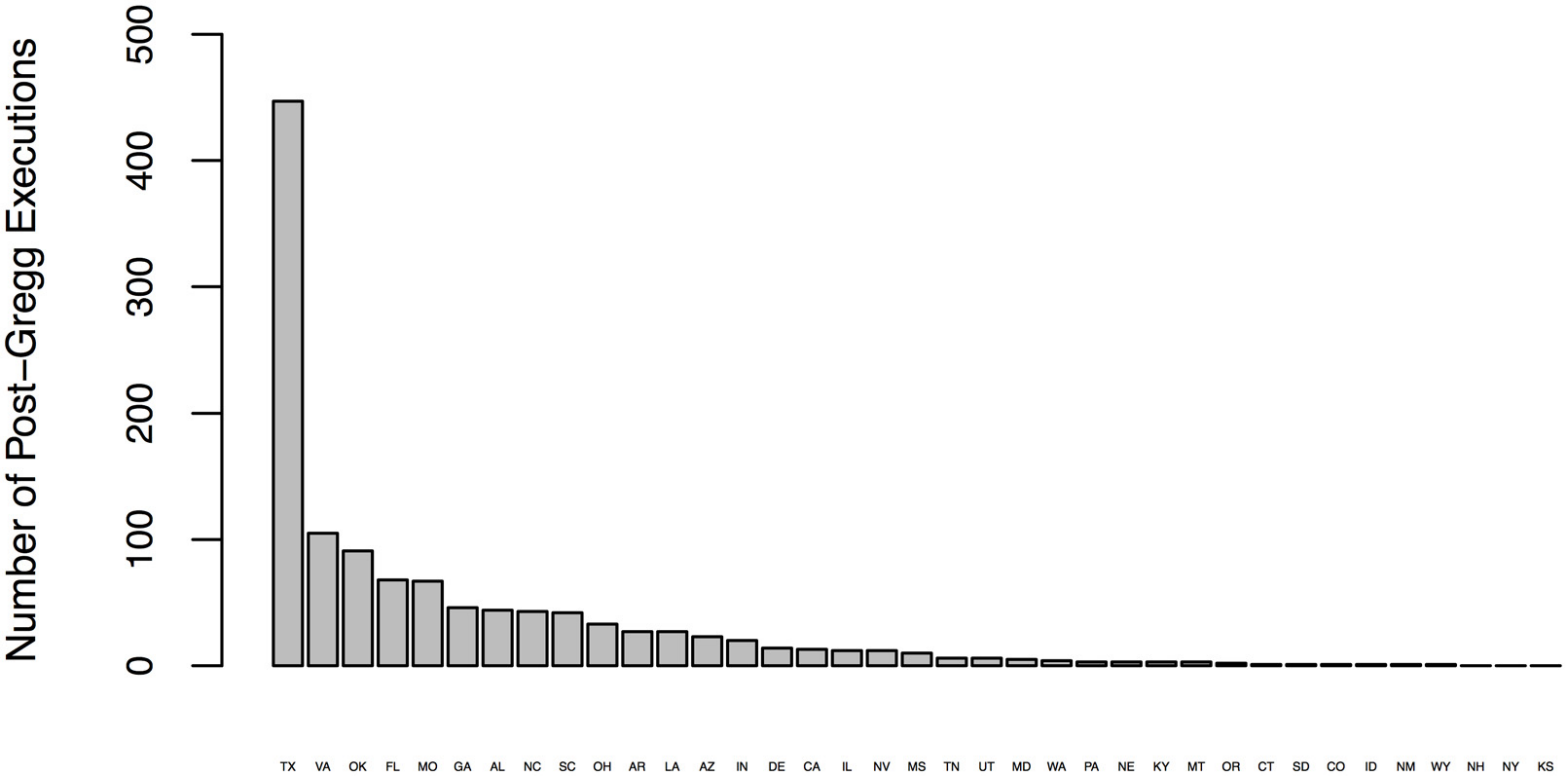
Count of Post-Gregg executions by state as of 2009.

[1] recently tackle another limitation of the modern panel studies of the death penalty: How to reliably identify the causal effect of executions on homicide given the sporadic, infrequent, or nonexistent application of executions in the majority of states with active capital punishment statutes. Fig 1 shows that of the 36 states with active death penalty statutes in 2009, only 13 have carried out more than twenty executions since the *Gregg* decision and 19 states have recorded 10 or fewer executions during this period [20]. What can also be inferred from Fig 1 is that most death penalty states in most years do not execute a single death row inmate. Berk’s [12] reanalysis of [4] reveals the highly skewed nature of the execution distribution in state panel data. Specifically, 86 percent of the state-year observations on the execution variable were equal to 0 with another 8 percent of the observations equal to 1. Importantly, only 5 percent of the observations contained execution values larger than 5. Given the relative infrequency of executions in the post-*Gregg* era, it is no surprise then that one of the main concerns raised by those assessing the sensitivity of the modern statistical evidence on the death penalty has been the suitability of panel studies when only a handful of states account for the lion’s share of the nation’s executions [1, 12, 14]. This is the main conclusions drawn by [13] after sifting through the recent death penalty literature: “Our key insight is that the death penalty at least as it has been implemented in the United States since Gregg ended the moratorium on executions is applied so rarely that the number of homicides it can plausibly have caused or deterred cannot be reliably disentangled from the large year-to-year changes in the homicide rate caused by other factors.” Berk’s [12] reanalysis of the state panel dataset of [4] shows that a few leverage points have unusually large effect on the conclusions about deterrence. Berk shows that their pro-deterrence findings were largely driven by 11 of the most influential observations (just 1 percent of the 1,000 state-year observations), with most of those observations occurring in Texas.

Not surprisingly, Land et al.’s [1] solution to this low dosage or insufficient treatment problem is to zero in on Texas, a state accounting for 464 of the 1,231 (38 percent) of all persons executed since the *Gregg* decision [20, 21]. They apply autoregressive integrated moving average (ARIMA) intervention time-series methods [22] to *first-differences* of monthly data on executions and homicides in Texas from 1994–2005. They build a series of seasonal linear transfer function models, using first and seasonal differences of both executions and homicides based on the assumption that “short-run” effects of executions needed to be separated from any “long-run” trends (or non-stationarity). Their decision to focus on the post-1993 period is based on U.S. Supreme Court decisions in *McCleskey v. Zant* and *Herrera v. Collins* which greatly limited *habeas corpus* petitions brought on by death row inmates in federal courts. They claim that these court decisions marked the beginning of a regime change in Texas since the number of death row inmates executed from 1994 through 2005, compared to the 1980 to 1993, grew by 300% (from 71 to 284) or from an average of 0.42 executions a month to nearly 2 executions per month. Therefore, the increased frequency and consistency of executions in the post-1993 period makes Texas a fertile test site for examining the deterrent effects of executions. [1] apply Granger causality tests to assess whether one or two-way causality exists between the two series and find no evidence of Granger feedback from homicide to executions. They build a series of seasonal linear transfer function models. Results for their best fitting model (see, their Table 2, Model 7) reveal a statistically significant reduction of 1.3 homicides in the first month following an execution with additional reduction of 1.2 homicides occurring 4 months later—for a total deterrent effect of 2.5 homicides per execution.

In our view, Land et al.’s [1] decision to first difference the execution and homicide series without formally testing for the presence of trends (be they deterministic, stochastic, or seasonal) leads to incorrect inferences about their key results. We argue and demonstrate that differencing possibly stationary or deterministically trending series such as executions and homicides induces false dynamics, making it wrongly appear that a causal relationship exists between the two series because of the false correlations. *Once we test for and properly model the data, the subsequent specifications are more parsimonious than those reported by [1] and strongly resist the interpretation that executions in Texas significantly deter homicides*. Perhaps more importantly, we show that the deterrent effects of executions are inflated and largely spurious because of the authors’ failure to control for other important factors coinciding with Texas’ execution binge and decline in homicides—the incapacitative effects of prison growth generally, and an improving economy (proxied with unemployment rates). The results of our analysis do not support the conclusions drawn by [1]. When controlling for changes in the number of persons incarcerated and unemployed, the magnitude of the effects of executions on homicide are less than half the published estimates and are statistically indistinguishable from zero. Consistent with previous research, we find that changes in incarceration were primarily responsible for reducing homicide after the mid-1990s. We employ the latest econometric time-series methods for interpreting time-series results, a set of impulse response functions with error bands that quantify the relative uncertainty of the effects of an execution on the number of subsequent homicides. Finally, we show that these results are robust to different, possibly endogenous changes in parameters using a series of changepoint models as a robustness test.

## Monthly Texas Homicides Time Series, 1994–2005

The Land et al. [1] claims about the deterrent effects of the death penalty on homicides in Texas relies upon a monthly analysis of the relationships of the executions on homicides. Based on data from 1994–2005, they conclude that the use of the death penalty in Texas reduces the short term number of monthly homicides by 2.5 based on ARIMA models with multiplicative seasonal processes. (We thank Ken Land and Hui Zheng for providing us their data.)

Like in most of the modern time series literature, we start with an initial characterization of the time series properties of monthly homicides in Texas. We do this so that one can then know the empirical regularities that need to be captured in building *any* model that purports to explain or predict aggregate homicides in Texas over recent decades. Despite the necessity to first analyze the dynamic properties of the time series of interest and then incorporate the effects of covariates, [1] rely on what we consider to be multiple *ad hoc* model specifications to assess the deterrent effects of executions on homicides. The end result is a set of atheoretical models that fail to account for the dynamic properties of the Texas homicide series and more importantly, induce spurious correlations with executions.

We start with an analysis of the presence of the deterministic trends, stochastic trends and seasonal trends or cycles in the homicides. This is part of the standard Box-Jenkins or dynamic regression framework. The initial goal of this analysis is to capture those parts of the time series of interest so we can separate out any potential correlates of homicide.

Critical to the specification analysis of these models are the determination of the presence of either trend or difference stationarity, with and without drift in the data. A deterministic trend stationary variable, *X*_*t*_ is a function of time, where the regression *X*_*t*_ = *βt* leads to a description of the trend. A difference stationary series is one where the first differences of the series of interest, say *X*_*t*_ is rendered non-trending by first differencing or using the *X*_*t*_ − *X*_*t* − 1_ = *Y*_*t*_ values of changes as the dependent variable of interest. One then proceeds to model these first differences as a function of their lags and a constant, such as $Y_{t}=c+\Sigma_{\ell =1}^{p}\phi_{\ell }Y_{t-\ell }$, where *c* would be the drift or the rate of change in the *X*_*t*_ series and the *ϕ*_ℓ_ coefficients capturing the remaining short run dynamics. If the trend model includes a constant, *c*, this is interpreted as a drift term which indicates the secular shift in the trend over time. Additionally, a time series may include both deterministic and stochastic trends or drift.

Determining the presence of trends is a critical part of ARIMA modeling. The analysis of [1] focuses on the short term effects, under the presumption that there was a stochastic trend in Texas homicides that needed to be removed. Here we show this is not the case. Specifying a trend or differencing the data when no stochastic trend is present leads to an incorrect ARIMA model and an incorrect estimate of the effects of executions on homicide reduction. We provide details on why this is the case below. We show how this can be corrected and provide proper inferences about this relationship. The results show that executions are not meaningful predictors of the reduction in the number of homicides in Texas. Further, we show that the [1] findings are a methodological artifact that is not robust to alternative dynamic model specifications and the inclusion of relevant correlates of homicide to mitigate omitted variable biases.

### Testing for trends, 1994–2005

To test for trends in the Texas monthly homicide series one can consider any of several tests in the literature. We start with the two of the more common: the augmented Dickey-Fuller (ADF) and the KPSS tests [23]. The ADF test assumes that the time series model is a random walk, or that the model of a time series *y*_*t*_ is

$$\begin{array}{c} y_{t}=c+\tau_{trend}t+\rho y_{t-1}+\epsilon_{t} \end{array}$$ (1)

where the coefficient *ρ* = 1 and the error process is Gaussian white noise. If we reject that *ρ* = 1, then we conclude that the series is stationary. The test is *augmented* by additional lagged values of the series to account for residual serial correlation. These additional lags are selected by a model fit statistic such as the Akaike Information Criteria (AIC). Further versions of this test can be constructed that test whether the deterministic trend coefficient *τ*_*trend*_ or *c* differ from zero. These versions allow one to rule out stochastic and deterministic drift in the monthly homicides time series.

The KPSS tests are parallels to the ADF tests [24]. Instead of evaluating the null hypothesis that *ρ* = 1, the null hypothesis is that the series is stationary or ∣*ρ*∣ ≠ 1. This test also has variants for whether a time trend is included in the model. As with the ADF test, there are short (4 lags) and long (13 lags) corrections to account for serial correlation.

Table 1 presents the results of a battery of ADF and KPSS tests applied to the 1994–2005 Texas monthly homicides series that are the main evidence in [1]. These tests have non-standard test statistic distributions that depend on the sample size and model specification; these are reproduced here for easy exposition. In total nine tests were computed to determine whether there is a trend in the Texas homicide data.

**Table 1:** ADF and KPSS tests for unit roots in the Texas monthly homicides, 1994–2005. Critical values given the right-most columns.

Test	Version	Statistic	1%	5%	10%
1	ADF, *ρ*_1_	-0.94	-2.58	-1.95	-1.62
2	ADF, *ρ*_1_	-4.14	-3.46	-2.88	-2.57
	ADF, *c*	8.61	6.52	4.63	3.81
3	ADF, *ρ*_1_	-4.44	-3.99	-3.43	-3.13
	ADF, *c*	6.74	6.22	4.75	4.07
	ADF, *τ*_*trend*_	10.07	8.43	6.49	5.47
4	KPSS, drift, short	1.17	0.74	0.46	0.35
5	KPSS, trend, short	0.54	0.22	0.15	0.12
6	KPSS, drift, long	0.53	0.74	0.46	0.35
7	KPSS, trend, long	0.26	0.22	0.15	0.12
8	KPSS, drift, none	3.75	0.74	0.46	0.35
9	KPSS, trend, none	1.53	0.22	0.15	0.12

Test 1 assesses the null of a unit root with no drift or deterministic trend using the ADF specification with no drift—or that *c* = 0 and *τ*_*trend*_ = 0. Based on the critical values in the right-most columns of the table, the test statistic of -0.94 does not reject the null hypothesis that the series is a unit root or trending variable. Note however that this model includes no drift or deterministic component. These are added in the ADF tests 2 and 3. In both tests the null hypothesis of a unit root is strongly rejected. The drift terms are significant as is the trend term. So we can reject the unit root hypothesis if we include a deterministic trend and/or a constant when modeling the series.

The KPSS results, Tests 4–9, use a different null hypothesis (stationarity and not a unit root, or *ρ* = 0 under the null), but come to the same conclusions. Tests 4 and 5 reject the null of stationarity, but with only a short lag correction for serial correlation. When longer lagged corrections for serial correlation are added, as in Tests 6 and 7, there is less evidence for rejecting stationarity. Finally, in Tests 8 and 9 where stationarity is assumed under the null with no corrections for serial correlation, the tests strongly reject the null of stationarity (in part because of the unmodeled serial correlation).

There is strong reason to suspect that differencing the data (both first and seasonal differencing) is incorrect. There is only weak to no evidence for non-stationarity or stochastic trends. In fact, the ADF and KPSS tests (Tests 2, 3, and 6) offer scant evidence for non-stationarity once a trend and/or constant are included in the model. So across the most general of the tests (2, 3, and 6) one sees equivalent results: there is no need to difference the data and there may be evidence of a deterministic trend. The reason to prefer these test results is that they are for more general null specifications that include different possible trends and serial correlation corrections. Failing to account for these alternative trends leads to potentially conflicting and erroneous inferences in the ADF and KPSS tests.

**Fig 2:**
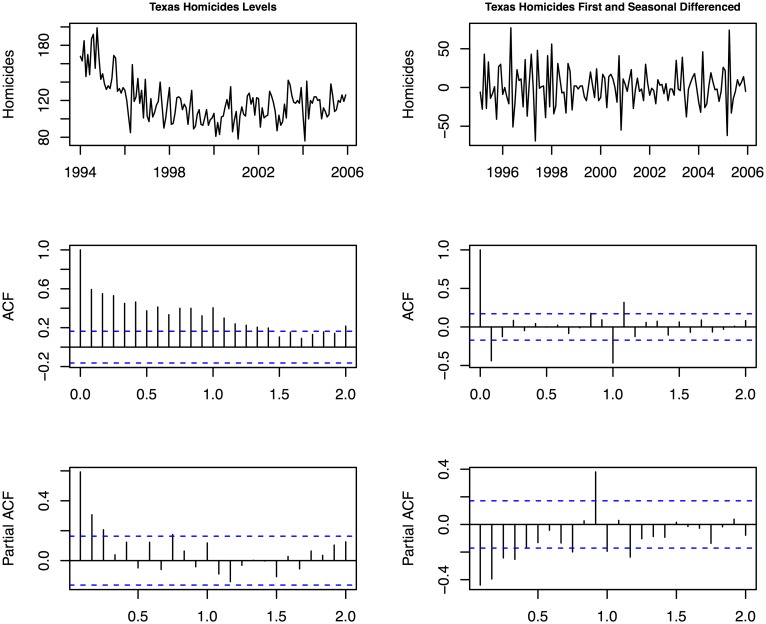
Texas homicides, first and seasonal differences and associated autocorrelation functions, 1994–2005.

We can graphically examine the data and some autocorrelations to see if there are trends in Texas homicides between 1994 and 2005. Fig 2 plots the raw data, the first and seasonal differenced data used in the [1] analysis and their associated autocorrelation functions (ACF) and partial autocorrelation functions (PACF). Column 1 (2) shows the data and correlations for the raw monthly (differenced) homicides time series. The ACFs show the raw correlations over different lags (measured in terms of the monthly period of the data) and the partial autocorrelations. The ACFs capture the pattern of raw correlations over lags *t* − 1, *t* − 2,…, and are used to assess the patterns of decaying autoregressive lags and moving averages. The PACFs capture the lags at higher lags controlling for those at lower lags, and are used to identify patterns of decaying and seasonal serial correlation. Data that are *overdifferenced* will exhibit serial correlation and moving averages at the period of the differencing [25].

The left (right) column of Fig 2 is the raw (differenced) data and correlation functions. The undifferenced series appears near stationary and has an ACF that decays slowly toward zero across 1.5 periods (18 months), indicative of a seasonally stationary process. The PACF for this series confirms this, with no significant lags after the first 12 months (period 1.0). The differenced series in the second column shows weaker to no autocorrelation but has statistically significant moving average spikes at lags 1 and 12 (at periods 0 and 1.0). These ACF and PACF results for the differenced series are consistent with those for a stationary series that has been overdifferenced. To see this, consider a stationary white noise time series process, or *y*_*t*_ = *ϵ*_*t*_ where *ϵ*_*t*_ ∼ *N*(*μ*, *σ* ^2^) (where the distributional requirement can be relaxed). If we first difference this series (regardless of the stationary dynamic process for *y*_*t*_) we see:

$$\begin{array}{ccc} y_{t}-y_{t-1} & = & \epsilon_{t}-\epsilon_{t-1} \end{array}$$ (2)

$$\begin{array}{ccc} \nabla y_{t} & = & \epsilon_{t}-\theta \epsilon_{t-1}, \end{array}$$ (3)

where we have assumed that *θ* = 1 and ∇ is a first differencing operator. So we have taking a stationary time series that may or may not have had a moving average component term and included one where *θ* = 1. This implies an autocorrelation function where the first order autocorrelation is $\frac{-\theta }{1+\theta }=-0.5$. So even if there is truly first order autocorrelation, it is not eliminated by taking this difference—it is just replaced by a spurious one.

The easiest way to see this time series identification point is to generate a series of independent and identically distributed or uncorrelated random variates and then apply a first and seasonal differencing to them. The resulting ACFs and PACFs will look like those in the second column of Fig 2 with PACF spikes of -0.5 at period 1. Since the generated data are serially uncorrelated, the resulting patterns are the result of overdifferencing the data. This is the same approach used to determine the order of differencing in non-stationary data, since when too many additional differences are taken, the result are excess moving average processes looking like those described here.

Based on these unit root tests, and the ACF and PACF results, it is very unlikely that Texas homicides over the 1994–2005 sample period should be modeled using first and seasonal differences, since there is no evidence of unit roots. In what follows we examine models for the undifferenced homicides’ time series since there is little to no evidence to support the approach taken by Land et al. Our assumption is that the series is stationary with possible changes in deterministic trends or regime.

### Identification, specification, and estimation, 1994–2005

We begin, with possible transfer functions for the the dynamics of the explanatory or input variables [22], analyzing the homicide series. This analysis begins with an investigation of the dynamics of the 1994–2005 Texas monthly homicide series. Based on Fig 2 and the earlier unit root tests, the initial model is a multiplicative seasonal ARIMA model with *p* autoregressive lags, *d* differences, *q* moving average terms, *P* seasonal, *D* seasonal differences, and *Q* seasonal moving averages. This is written as an *ARIMA*(*p*, *d*, *q*)(*P*,*D*,*Q*)_*s*_ model where *s* is the period of the seasonality, in this case *s* = 12. Since the last section offered no evidence to support differencing, we set *d* = *D* = 0.

The initial model for the Texas homicide series *h*_*t*_ sample is an *ARIMA*(3,0,0)(1,0,1)_12_ with a deterministic linear trend *t*. This model was selected based on the results of the earlier unit root tests which indicated that there was no stochastic trend (so no need for first differencing), but the possibility of a deterministic trend as a function of time. The ACF and PACF functions provided an initial idea of the serial correlation patterns and lags to be used. These were then refined until white noise residuals were achieved based on Box-Ljung statistics for correlations at various lags up to 16 months. The results of this estimation strategy are (with standard errors in parentheses):

$$\begin{array}{ccc} h_{t}=\underset{\left(17.5\right)}{146.7} & + & \underset{\left(0.16\right)}{-0.29}t+\underset{\left(0.08\right)}{0.34}h_{t-1}+\underset{\left(0.08\right)}{0.13}h_{t-2}+\underset{\left(0.08\right)}{0.32}h_{t-3}+\underset{\left(0.10\right)}{0.93}h_{t-12}\\ & + & \epsilon_{t}-\underset{\left(0.20\right)}{0.76}\epsilon_{t-12}. \end{array}$$ (4)

The Box-Ljung statistics for residual serial correlation in indicate no unmodelled serial correlation for lags one through 16. This is an ARIMA model with several interesting features. First, the sum of the first three autoregressive terms is stationary since ∣0.79∣ < 1 and all of the coefficients are positive. The fact that this sum is less than 1 is indicative of a stationary process. The ARIMA results are consistent with the earlier unit root tests and provide evidence that the homicide series should not be first or seasonally differenced prior to analysis. Second, the deterministic trend term *t* has a p-value of 0.03 or a significant negative trend. This captures the downward trend in the number of Texas homicides over the sample: the mean number of homicides per month is 1994 is 169 while it is 117 in 2005. Third, the seasonal component is stationary with a seasonal AR term of 0.93. A seasonally differenced version of this model is plagued by serial correlation and fits worse than this model. This alternative model’s Box-Ljung statistics indicate serial correlation after lag three. The error variance of the estimate for the seasonally differenced model is 231; it is 224 for the non-differenced data model in Eq (4).

Since this model has no residual serial correlation it is a good basis for inference about the Texas homicides. Any efforts to model this series as a function of other variables has to have them mimic the properties seen in this basic ARIMA model: there is positive serial correlation, strong seasonal effects that decay slowly, and a shift over time in the trend.

### Transfer function analysis, 1994–2005

One of the most difficult challenges facing death penalty researchers using either a single time-series or panel designs concerns specifying the timing for when changes in execution risk are supposed to have an effect on the number of homicides. Should changes in execution risk have an immediate or lagged effect on homicides? While it may be difficult in many cases to specify when a change in law or policy is supposed to have an effect on the target variable, in those instances where theory (or an understanding of the intervention) does provide guidance on the matter, it is imperative those candidates be selected and tested [26]. Failure to do so can result in making *ex post facto* interpretations (which may be true) for unexpected results, thereby making the policy efficacy hypothesis nearly impossible to falsify [27]. As noted over a half a century ago by [28]: “If such time series are to be interpreted as experiments, it seems essential that the experimenter must specify *in advance* the expected time relationship between the introduction of the experimental variable and the manifestation of an effect” (emphasis added).

The most common intervention point in the single time-series or panel design death penalty literature is a one time period lag of the execution risk measure under the assumption that it takes time for actual changes in execution risk to alter prospective killers perceptions of execution risk. However, we are not aware of any compelling explanation in the deterrence literature as to why it would take several months or up to a year for prospective killers, whether assessing risk consciously or unconsciously, to be reminded of the possibility of being executed for murder. Land et al. [1] take another approach in the time series policy literature, relying on ARIMA model building methods to identify the best-fitting model (i.e., let the execution and homicide data speak for themselves). They include all lags of executions 1 to 12 in the homicide models and, as noted above, find that the best fitting ARIMA model is one which produces statistically significant coefficients for executions at lags 1 and 4. They interpret the significant coefficients for executions as a deterrent effect of the death penalty and provide a statistical explanation for why the deterrent effect of executions ceases to exist after one month and then picks up again three months later. As far as we know, there is nothing in the deterrence doctrine or any other criminological theory that can even remotely explain the impact patterns they have identified. Put another way, the belief that prospective killers consider execution risk in the subsequent month following an execution, cease to consider risk of execution two and three months later, but then consider risk of execution some four months later is implausible at best, impossible at worst.

We suggest the more theoretically defensible model, within the deterrence framework, is to estimate a dynamic input model that adds contemporaneous and once lagged counts of the numbers of executions as predictors in the homicide model of Eq (4). To the extent that deterrence depends on conscious consideration of risk, more frequent and recent executions should serve as the greatest reminder to prospective killers of the risk of execution. Conscious assessment of risk does not have to be very sophisticated, and thus does not even necessarily entail comparing execution frequency from one month to the next. Conversely, preconscious considerations of execution risk, especially those rooted in fear, might also be influenced by places and times with more frequent executions. While it is impossible to know for sure how execution frequency is treated by prospective killers in risk assessment calculations, assuming they are considered at all, we believe this model offers the most plausible scenario by which deterrent effects via frequent executions is likely to manifest itself.

Table 2 presents the results for the ARIMA model with the contemporaneous and lagged monthly number of executions included in the model. The basic ARIMA model specification is as in Eq (4) and is presented in the first column of Table 2. The only change here is the addition of the regressor(s) for the number of executions. In the table, column 1 reproduces the baseline ARIMA model for the homicide series with *ϕ*_*j*_ as the autoregressive coefficient for the *j*′ *th* lag, *t* is the deterministic linear time trend and *θ*_*k*_ is the *k*′ *th* lag moving average coefficient.

**Table 2:** ARIMA transfer function models of Monthly Texas Homicides, 1994–2005. Standard errors in parentheses.

Intercept	147	148	149
	(17.5)	(16.6)	(15.4)
*t*	-0.29	-0.29	-0.28
	(0.16)	(0.15)	(0.15)
*Executions*_*t*_		-0.93	-0.90
		(0.68)	(0.68)
*Executions*_*t* − 1_			-0.82
			(0.69)
*ϕ*_1_	0.34	0.32	0.32
	(0.08)	(0.09)	(0.09)
*ϕ*_2_	0.13	0.15	0.15
	(0.08)	(0.08)	(0.09)
*ϕ*_3_	0.32	0.31	0.30
	(0.08)	(0.08)	(0.09)
*ϕ*_12_	0.93	0.92	0.90
	(0.10)	(0.12)	(0.14)
*θ*_12_	-0.76	-0.75	-0.70
	(0.20)	(0.22)	(0.25)
*σ* ^2^	223.7	221.5	222.2
LLF	-596.95	-596.02	-591.68
AIC	1209.89	1210.04	1203.36
*R* ^2^	0.58	0.58	0.57

The contemporaneous effect of executions on homicides is very small. The effect of contemporaneous executions on homicides is −0.93 with a standard error of 0.68. The two-sided p-value for whether this contemporaneous effect in column 2 of Table 2 differs from zero is 0.17. The p-value of the joint effect of the contemporaneous and lagged effects modeled in column three of Table 2 is 0.20. The total effect or cumulative drop in the number of homicides is given by the impact multiplier

$$\begin{array}{c} \frac{\beta }{1-\phi_{1}-\phi_{2}-\phi_{3}}=\frac{-0.93}{1-0.31-0.15-0.31}=-4.28. \end{array}$$

So there are four fewer homicides after an execution. The estimate for the model with two lagged values of executions is -7.3 fewer homicides. Repeating the earlier impact multiplier computation for the model with the contemporaneous and lagged executions as regressors yields $\frac{-0.90-0.82}{1-0.32-0.15-0.30}=-7.3$. These estimated effects are larger than those reported in misspecified ARIMA models in Land et al.

It bears emphasizing, however, that substantively these estimates are very small and uncertain. The standard deviation of the residuals (reported in Table 2) is on the order of 15 homicides. So even the largest effect is within a standard deviation of the residual error of the model. Our argument, however, is that the effects of executions on homicides are highly uncertain and substantively meaningless. There are three reasons for this claim.

**First**, the p-values for the hypothesis tests for whether the coefficients for the executions variables are different from zero are barely different from the widely and erroneously accepted 5% level of significance. Given the possible model uncertainty and the restriction of the sample to 1994–2005, we should require more significant evidence, even for a one-sided hypothesis test.

**Second**. these marginal and total effect estimates do not fully account for the uncertainty of the parameters in the ARIMA specification. Accounting for this full uncertainty requires the computation of an impulse response and its error bands. The impulse response traces out the impacts of a one unit change in the number of executions on the dynamic path of homicides. This can be accomplished via a dynamic simulation of the ARIMA model that accounts for the model uncertainty for a single increase in the executions given that the parameter estimates equal those in Table 2 with error variances ${\hat{\sigma }}^{2}$ as reported in the table. This is consistent with the time series econometrics literature described in [29] and [30] who document that the best practices for tracing out the dynamic effects of a change to a covariate or a unit shock in a time series model is via a Monte Carlo simulation. Such a simulation (conducted below) accounts for the parameter uncertainty and the sample size of the model when estimating the *distribution* around the change in executions on the number of homicides over time.

**Third**, estimating the impulse response function for the effect of a marginal change in executions on the number of homicides per month will allow us to measure and report the possible asymmetry in the effects. The reason this matters is that a decision-maker’s cost function about this research is clearly not symmetric. In the decisions and inferences about deterrence, the cost of a Type I error is not likely to be the same as a Type II error. More bluntly, the cost of an execution relative to a potentially saved life is not the same. The way we can help quantify this tradeoff is to provide information about the full density around the impulse response function which traces out the dynamic impact of a single execution on the homicides series.

To address these concerns, we simulate the impulse responses. This allows us to see both the uncertainty around the effects and over what horizon the responses are observed. An impulse response (function) traces out a path of the homicides assuming one additional execution. With impulse responses one is interested in the size and uncertainty or variance around the response as well as how long it takes for the effects to be realized, based on the estimated time series model. The impulse response effectively shows the time path of the cumulative response or impact multipliers computed above. More importantly, we want to look at the shape and variance of the response. These allow us to assess the likelihood that an execution affects the number of subsequent homicides—an important first step in establishing the deterrent effect of executions.

**Fig 3:**
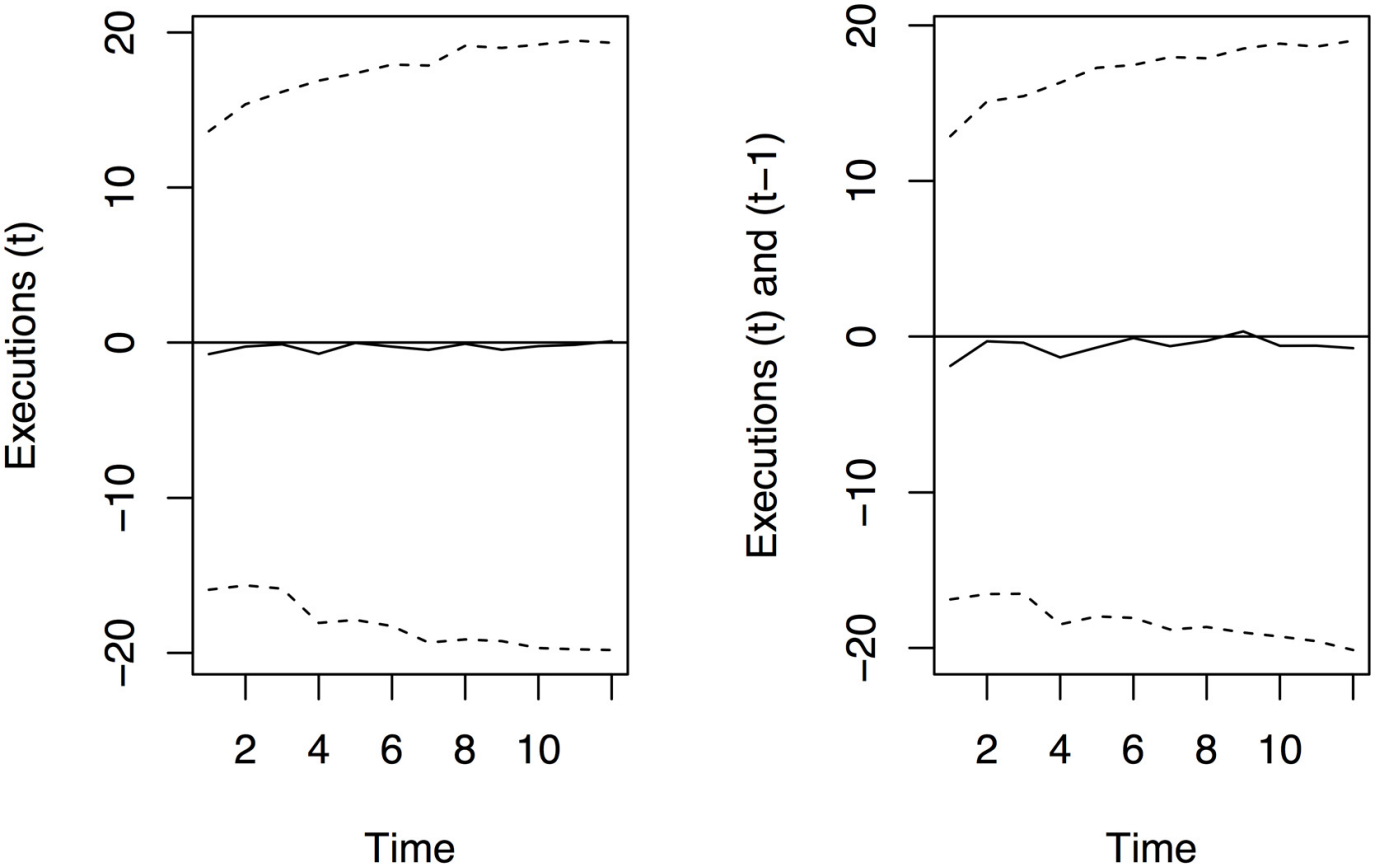
Impulse Response Functions for One Execution for Models in Table 2. Responses are in solid lines with 68% or approximate one standard deviation confidence regions in dashed lines.

Impulse responses are best summarized using Monte Carlo simulation since this allows one to construct an error band or standard error estimate around the dynamic path of the effect of an execution [29]. The impulse responses for the effects of executions from the last two models in Table 2 were simulated 5000 times and are summarized in Fig 3. These responses account for both the ARIMA regression parameter uncertainty *and the residual variance of the responses over 12 months*. Failing to account for the residual variance uncertainty in the simulation yields identical modal impulse response estimates. But it will lead to understating the uncertainty by a by nearly a factor of 20 because it fails to model the uncertainty from the estimate of *σ* ^2^ for each model. Once this residual parameter uncertainty is included (correctly) in the Monte Carlo simulation, the resulting estimates are those in the figure. The modal estimates are presenting using the solid line while the 68% confidence region, or approximately one standard deviation confidence region of the response is plotted using dashed lines.

These impulse responses trace out the change in the number of homicides for an additional execution based on the models in the final two columns of Table 2. As the figures show, the total estimates over twelve months match those predicted: within 12 months the first transfer function model predicts 3.34 fewer homicides (the cumulative sum over the 12 months, out of a total of 4.28 fewer) and the second transfer function model predicts 7.02 fewer homicides (the cumulative sum over 12 months, out of a total of 7.3 fewer). But these effects are not statistically or substantively meaningful because the error bands on the responses cover zero over the full time horizon. So while there is an effect, it is swamped by the residual variation in the monthly Texas homicide series. We also computed Bayesian-shape error bands that account for the serial correlation in the uncertainty of the impulse response estimates [29]. While these error bands are smaller than those in Fig 3 the same conclusions still hold.

The conclusion here is straightforward: there is no evidence in this analysis or that of [1] that can be used to offer evidence for executions having a policy-relevant, deterrent effect on homicides in Texas over the 1994–2005 period. As a preliminary conclusion, the results of [1] are highly sensitive to model specification assumptions.

## Another model

Based on the estimates in Table 2 and the impulse responses in Fig 3 the estimated effects of executions do not have a deterrent effect on the number of monthly homicides in Texas. Our claim is based on the fact that once we account for the residual variation of the fitted ARIMA models, it is hard to say that the effects of executions on homicides are substantively meaningful. This is because the ARIMA model only accounts for about 58% of the observed variation in the homicides series, per Table 2.

So one might ask, how could a better fitting time series model be constructed? Could the the effects of execution be more clearly revealed? This is mainly a call to advance inquiry beyond the bivariate analysis to the inclusion of relevant controls to reduce the risk of omitted variable bias and spurious inference for the execution variable. Such a criticism has been made recently by [25].

To evaluate this possibility, we specified additional models that included two additional covariates. The first is the (monthly) number of tens of thousand of prisoners in Texas prisons. These data on the on-hand offender population by month were obtained from the Texas Department of Criminal Justice (TDCJ) through a public information/open records request on September 3, 2010. Several studies such as [31] and [32, 33] suggest that controlling for prison expansion in the U.S. and Texas is a necessary covariate for analyzing the drop in crimes seen across the U.S. in this period. During the study period covered by [1] (i.e., 1994 to 2005), the number of people incarcerated in Texas prisons increased dramatically from 62,322 to 151,925 or by 143 percent. A sophisticated multivariate panel analysis of Texas counties finds that most of the drop in violent and property crime in Texas during the 1990s can be attributed to increases in the number of offenders in jail and prison [34]. It is worth noting that [34] explicitly controlled for executions in both the violent and property crime specifications and found no evidence of a deterrent effect of executions on either crime type at occurring at the county-level. Of course, the results for the execution variable may have differed if homicide had been used as the dependent variable. [32, 33] also cites prison growth as one of the leading causes of the crime decline, especially for homicide, experienced in the U.S. during the 1990s [31, 35, 36].

The second covariate is the first differences of the Texas unemployment rate. This monthly-level unemployment data for Texas were obtained from the Bureau of Labor Statistics website on April 18, 2012. We use differences for the unemployment data because they have a unit root. Failing to do so would lead to an unbalanced specification with a trend, or a spurious regression. Most macro-structural theories of crime contend that improving economic conditions should result in less crime, although the relationship between unemployment and crime is admittedly tenuous, at best [37–39]. Many of these same theories, however, predict a criminal justice system that is less punitive when the economy is improving as unemployed/marginal workers are perceived as less threatening and needed potential laborers [40–43]. This was clearly not the case in Texas or anywhere else in the U.S. throughout most of the 1990s and early 2000s. Indeed, in most years between 1994–2005 unemployment rates were lower than the previous year’s levels while incarceration rates and the number of persons executed were generally higher than the previous year. This means the deterrent effect identified by Land et al. may be a spurious correlation since Texas’ decision to increase the use of the death penalty coincided with an improving economy.

Again, we restrict the analysis to the critical 1994–2005 time period for comparison to the results in [1]. We use the earlier ARIMA specification and look at the effects of adding these additional covariates on the results. Table 3 presents the results with these new covariates. The effects of an additional execution are smaller here: the estimated coefficient is now −1.31, but the dynamic multiplier is $\frac{-1.31}{1-0.14}=-1.52$. So the effects of additional executions are mitigated when we control for the number of prisoners (in 10000s) and the change in unemployment. Each additional 10000 prisoners lowers the number of homicides by nearly 9, or a total effect of 10.4 fewer homicides. So changes in incarceration alone predicts a larger drop in homicides than executions. Also, the effect of changes in unemployment is not a predictor of homicides in Texas. Note also that this last model is superior to second column of results in Table 2, since a likelihood ratio test comparing them has a value of 18.2 (p-value < 0.01). We also considered specifications using changes in the number of prisoners month-over-month and year-over-year. Neither of these rival specifications fit as well as that reported here.

**Table 3:** ARIMA transfer function models of Monthly Texas Homicides with additional covariates, 1994–2005. Standard errors in parentheses.

Intercept	237
	(11.7)
*t*	0.096
	(0.054)
*Executions*_*t*_	-1.31
	(0.70)
*Prisoners*_*t*_	-8.96
	(1.02)
∇*Unemployment*_*t*_	1.03
	(5.17)
*ϕ*_1_	0.14
	(0.09)
*ϕ*_2_	-0.03
	(0.09)
*ϕ*_3_	0.09
	(0.09)
*ϕ*_12_	0.97
	(0.06)
*θ*_12_	-0.83
	(0.17)
*σ* ^2^	192.1
LLF	-586.92
AIC	1195
*R* ^2^	0.64

The results of this section confirm a rather obvious result: there are omitted variable biases in the ARIMA specification. That is, we can easily improve the fit of the homicide time series model in Eq (4) by adding strongly suggested covariates such as the change in the number of prisoners. As in most omitted variable problems, the marginal effects of substantively significant variables changes radically when different specifications are examined. So adding a variable that a) is predicted to negatively be correlated with the homicides and b) positively correlated with executions (since more prisons mean there can be more people sentenced) will attenuate the effect of executions. The number of monthly homicides is correlated with the number of 10000 prisoners at −0.7 and the number of executions is correlated with the number of 10000s prisoners at 0.19.

Simply put, bivariate time series studies of the death penalty, no matter how statistically sophisticated, are virtually worthless for evaluating the deterrent effect of executions as they cannot rule out any alternative explanations for changes in homicide, a point recently and cogently made by [25].

## Changepoint analyses

Possible causes of the sensitivity of these results are that there are time-varying or omitted structural changes in the homicides series and the other variables. These could include the sanctions regime discussed in [25]. The issue is that given the discussion in [1], we are unsure of when the sanction regime changes in Texas. One way to evaluate the possible sanctions regime effect is to employ a model that allows for structural changes in the regression parameters. The Bai and Perron (1998) [44] model is commonly used to detect such structural changes. The model assumes a regression with *m* structural breaks (or *m* + 1 regimes) with the following specification

$$\begin{array}{c} y_{t}=x_{t}^{\prime }\beta +z_{t}^{\prime }\delta_{j}+u_{t},\;t=T_{j-1}+1,\ldots T_{j} \end{array}$$

for *j* = 1,…,*m* + 1. Here, the dependent variable time series *y*_*t*_ is explained by a set of fixed regressors and a set whose effects change over the *m* + 1 regimes. The effects of regressors *x*_*t*_ are not time-varying, while the effects of *z*_*t*_ do depend on the changepoints or regimes. The indices for the breakpoints are *T*_1_,…,*T*_*m*_, and define when the changepoints occur (assuming that *T*_0_ = 0 and *T*_*m* + 1_ = *T*). The timing of the breaks or the values of the *T*_*j*_ are assumed unknown and need to be estimated.

For each partition of the sample into (*T*_1_,…,*T*_*m*_)—meaning a split of the sample into the *m* + 1 regimes—we minimize the sum of squared errors,

$$\begin{array}{c} S_{T}\left(T_{1},\ldots ,T_{m}\right)=\sum_{i=1}^{m+1}\sum_{t=T_{i-1}+1}^{T_{i}}{\left[y_{t}-x_{t}^{\prime }\beta -z_{t}^{\prime }\delta_{i}\right]}^{2} \end{array}$$

The estimator then selects the optimal split of the sample into the estimated breakpoints $\left({\hat{T}}_{1},\ldots ,{\hat{T}}_{m}\right)$ that minimizes the sum of squared errors. The optimal number of breakpoints is selected based the minimum the Bayesian Information Criteria (BIC) that accounts for fit, but penalizes using too many parameter or regimes. We examine dynamic regression model specifications with zero to five breaks and report those that minimize the BIC for a given specification of regressors.

We begin with an admittedly underspecified breakpoint analysis of the full monthly homicides series, covering from 1980(1)–2009(8). For this series, regressed only on a constant (so this is a simple changing means model), the optimal number of breakpoints is three, at 1984(12), 1990(4), and 1994(11) (results not reported). One problem with this initial specification is that it does not capture the autoregressive or seasonal components of the homicides series. Note the Land et al. sample comes after each of these breakpoints. But this sets a simple upper bound on the number. Including covariates for the dynamics of the homicides, the number of prisoners, the number of executions, etc. should explain some of these breakpoints or change the dates of their estimated locations.

Table 4 presents the dynamic regression model with one break. This two regime model optimally splits the sample at 1994(10) and accounts for the autoregressive dynamics of the homicides series (the residuals for this model are white noise). Note that the intercept drops in the latter period (after 1994) when the number of homicides falls. Also, the AR(1) process weakens (the coefficient drops from 0.42 in the first regime to 0.20 in the second regime).

**Table 4:** Changepoint model for monthly Texas homicides, 1980(1)–2009(8). First entry is the coefficient, second in parantheses are the standard errors, followed by the two-sided p-values.

	Regime 1	Regime 2
Periods	1990(1)–1994(10)	1994(11)–2009(8)
Intercept	56.282	50.810
	(12.091)	(9.693)
	< 0.001	< 0.001
*Homicides*_*t* − 1_	0.421	0.195
	(0.055)	(0.087)
	< 0.001	0.025
*Homicides*_*t* − 12_	0.272	0.351
	(0.054)	(0.071)
	< 0.001	< 0.001
*R* ^2^	0.986	
*σ* ^2^	18.377	

Next, we include the covariates in the breakpoint model. We include here the year-over-year percentage change in prisoners (since the levels of prisoners are possibly non-stationary) and the number of executions at time *t* and *t* − 1, as before. We also looked at models with only the change in prisoners or only the executions, which produce similar results. We only have the prisoners’ data from 1985(3), so the sample for the change specification (year-over-year) begins in 1986(3). The optimal breakpoint model with this specification is reported in Table 5

**Table 5:** Changepoint model for monthly Texas homicides with covariates, 1986(3)–2009(8). First entry is the coefficient, second in parentheses are the standard errors, followed by the two-sided p-values.

	Regime 1	Regime 2
Periods	1986(3)–1992(1)	1992(2)–2009(8)
Intercept	14.129	31.475
	(17.136)	(6.430)
	0.410	< 0.001
*Homicides*_*t* − 1_	0.437	0.300
	(0.075)	(0.069)
	< 0.001	< 0.001
*Homicides*_*t* − 12_	0.457	0.442
	(0.091)	(0.057)
	< 0.000	< 0.000
%∇*Prisoners*_*t* − 12_	1.650	0.010
	(0.473)	(0.114)
	0.001	0.927
*Executions*_*t*_	6.891	-0.610
	(3.353)	(0.729)
	0.041	0.404
*Executions*_*t* − 1_	-3.261	-0.551
	(3.406)	(0.732)
	0.339	0.452
*R* ^2^	0.986	
*σ* ^2^	17.369	

The optimal number of breakpoints for this specification is one and it splits the sample at 1992(1)—prior to the date of Land et al.’s sample. The dynamics of the homicides series (seen in the AR coefficients) are remarkably similar before and after the breakpoint. Note that the effects of the main covariates are contrary to expectations: executions increase the number of homicides prior to 1992 and are not statistically significant predictors after 1992. This further supports that argument that the specification decisions and lack of structural identification make it difficult, if not impossible to make clear claims about the relationships between executions and homicides in Texas over this sample period.

## Discussion

Our conclusions about the effect of executions on homicides in Texas from 1994–2005, as reported in [1] are very sensitive to model specification decisions and samples.

First, changing from a model of first and seasonal differences to one with a pure autoregressive specification leads to one that is more parsimonious and better explains the dynamics of the Texas monthly homicides over this stretch. This is critical, since without a correct dynamic specification of the basic ARIMA process, the subsequent analysis of the effects of executions on homicides will be incorrect.

Second, using the improved time series specifications, we see that the results about the effects of executions are very sensitive to model specification decisions vis-a-vis those made in [1]. When we change the dynamic specification we get an estimated effect that is larger than that reported previously, but also more uncertain. Using a standard interpretative method to assess the dynamic effects, an impulse response function with error bands to ascertain the full uncertainty of the model and its estimates, indicates that the effects of executions on homicides are no different than random chance. So while the impact multiplier is negative and weak evidence for the executions deterring homicides in Texas, the results are not unambiguous.

Third, the results are called into question when we add two highly plausible covariates to the analysis: changes in unemployment rates and the number of prisoners in Texas prisons. Adding these covariates to the analysis shows that the incarceration of more criminals predicts a larger drop in the number of Texas homicides than the effect predicted by an execution. But even with these additional controls, the large residual variance swamps the estimated dynamic effects. So even for this updated model, there is little to no evidence that strongly suggests that either incarceration or execution changes substantively the number of homicides in Texas between 1994 and 2005.

Fourth, the changepoint model results in the previous section show that the critical changes come prior to 1994 and in fact do not work as Land et al. suggested. Rather we see that the effects for executions are weak and not significant by any conventional expectation. Further, the lack of identification makes determining the effects of these deterrence and incapacitation variables quite hard.

What substantive conclusions should be drawn from these results? The initial conclusion should be that research purporting to find effects for executions on homicides are likely suspect because of issues involving model underspecification and interpretation. One typically likes to interpret the marginal impacts of single covariates, but this alone does not account for all of the full dynamic effects or the potential model misspecification. Using the impulse analysis methodology employed here does a much better job of this.

Further there is no good identification strategy for disentangling the possible deterrent effects of executions on homicides [25]. At best, what is presented here suggests that there might be some weak correlations. At worst, it shows that the inclusion of different covariates or model specifications lead to dramatically different and highly sensitive results. This is not a good basis for making policy projections or decisions since there is no evidence that the effects of the covariates like executions on the homicides variable are robust to different specification or identification assumptions. While one can continue to suggest new or additional covariates, the limited degrees of freedom for the homicides series means that there need to be more theoretical work ahead that can define better data analysis strategies.

Finally, we caution that these results are based on what can be described as good, but not exhaustive time series data analytic techniques. Employing ARIMA and changepoint models we are making explicit assumptions of the endogeneity of the homicides series and the exogeneity of the other covariates (the input series). This is typically hard to defend in many social science applications [45]. So future empirical examinations should employ methods that look at the possible dynamic, endogenous nature of the aggregate homicides time series with other predictors, rather than making indefensible strict exogeneity assumptions.

## Supporting Information

S1 Replication Archive Replication datasets and R scripts for all analyses in a zip archive. (ZIP)
